# Supplementary material for: Associations between environmental factors and running performance: An observational study of the Berlin Marathon
Source: PLoS One. 2024 Oct 16;19(10):e0312097. doi: 10.1371/journal.pone.0312097 (PMC11482731; doi:10.1371/journal.pone.0312097)
Supplement: Attachment — Submitted filename: PONE-D-24-15986_Revision 1_Comments.docx [file pone.0312097.s001.docx]

**Editor:**

Please note the followings when you revise the manuscript. The manuscript is required to improve writing. Specifically, the manuscript does not have a central focus that you would like to present to the reader. In the discission section, main hypotheses and findings are presented in the first paragraph but not fully discussed or compared to the previous literature. Also, there is limited physiology used to explain the results or why you were investigated to begin with. The manuscript may be of benefit to the literature but in its current form it is hard to fully grasp the importance of the data.

**Answer:** We thank you for the thorough review and have made an effort to address all stated issues. We have reworked our discussion section and curtailed our figures to present a more focused and readable manuscript while highlighting the important results of our study.

**Reviewer #1:**

The manuscript titled “Associations Between Environmental Factors and Running Performance in Age Group Runners: An Observational Study of the Berlin Marathon” found that increasing environmental temperatures negatively impacted the overall running speed. The examination is unique due to the large data set (520, 521 men, 147,988 women), the multiple environmental parameters, and extensive statistical analyses. The analyses and discussion, while interesting, were difficult to wade through. Specifically, there was some disconnect between the main hypotheses, analysis presentation, and discussion of these findings. I have provided comments in the hope that it will streamline the main findings and bring the importance of these analysis to the forefront.
**Answer:** We agree with the expert reviewer and have addressed these concerns as best as we could.

Comments
Title: The title does not reflect the analyses. The title includes “age group” runners, but no analysis on age was completed. Please reword.
**Answer:** We agree with the expert reviewer and have amended the title and removed the term age group.

Abstract: Please reconsider your use of the term “Recreational Runner”. Runners completing the marathon in 2:30 would still display a V02 in the 90% for the population, especially is they were female, and would have likely taken months of regimented training to accomplish this time.

**Answer:** We have changed the term to amateur runners to better classify these participants who do not receive monetary compensation for participating. We agree that the outliers who fall into the under 2:30 group are exceptional runners who have prepared for these events for months.

The conclusion that these results could be used to “manage hydration and running tempo” was not addressed anywhere in the discussion.

**Answer:** We agree with the expert reviewer and have aligned the conclusion statement in the abstract with the one in the conclusion section.

Additionally, your analysis was a retrospective analysis on how running speed may have changed with environment and there was no predictive effect on pacing. This fact should be considered when presenting the results in the discussion as well.
**Answer:** We agree with the expert reviewer and have highlighted this as suggested.

Introduction: Please double check references. Many references used within the paper apply to multiple statements but are excluded, specifically around past examinations of marathon performance and environmental conditions. A reference which may assist in the analysis of cloud cover and solar load (PMID 17986912). The physiology presented in lines 102-112 only address part of heat production and does not appropriately address heat dissipation and why the environment would be of importance. Please see and reference PMID 13930723. **Answer:** **Answer:** We agree with the expert reviewer and have included the suggested references in the manuscript.

Line 114-121: the majority of the introduction is about environmental temperature, but you mention that you want to investigate other environmental factors such as barometric pressure, please provide greater rational for investigating these other factors.
**Answer:** We agree with the expert reviewer and have added a greater rationale to our Introduction to address this shortcoming.

Methods: Please identify if you are using Relative or Absolute humidity. There is a correlation between absolute humidity and heat dissipation, less so for relative humidity. Did you account for improved performance with time.

**Answer:** We have used relative humidity in our analysis, as highlighted by the use of percents as a unit instead of g/m^3^. As for the improved performance with time, we could not account for the participant’s acclimatization because we don’t have the data from which we could derive it.

As you mention in line 89, Berlin has had the most world records. Clearly there is a shift to increasing times for all athletes for the past 24 years.

**Answer:** Indeed, the pacing strategies of the best marathon runners in the world have changed over the last 50 years, as stated in the referenced paper doi: 10.1080/17461391.2018.1450899.

Did you account for course topography when calculating pacing and comparing between each 5K?

**Answer:** We did not account for the course topography since the Berlin Marathon features a flat course.

Did you obtain humidity from the Deutscher Wetterdienst? It is not listed in lines 148-151. **Answer:** Yes. We have forgotten to add it. Thank you for the hint. We have amended our manuscript.

Did you average the hourly weather parameters for the length of your time grouping? If so, how did you account for hourly weather data, yet had time groups of 30 minutes?
**Answer:** We have classified all race records in 30-minute groups, and then the average values of the weather variables for each group were calculated according to the following scheme:

It seems that we made an error in our calculation. For the sunshine (minutes) and precipitation (mm) variables, rather than averages we should have calculated cumulative values over the race time. We have corrected our calculation accordingly.

Results: There are extensive results and figures that do not address the main hypotheses presented by the authors.

**Answer:** We agree with the expert reviewer and have removed some of the unnecessary figures.

For example, the average running speed of all athletes was 10.5 km/hr and figure 1 shows average running speed by all runners per year, per 5K but weather is not incorporated, nor is there error or variability presented.

**Answer:** We have tried to improve the readability of these charts by removing the shading and using a more colorblind scheme including dashed, dotted, and full lines. We also added the series for the final spurt (called “42K PACE”) which shows some interesting observations.

There is so much data presented in multiple ways, it is unknown what the authors would like the reader to focus on, and/or what data directly address the authors stated hypotheses. Were men’s and women’s changes in pacing directly compared? These seem to be separate analyses.

**Answer:** We agree with the expert reviewer and have removed some of the unnecessary figures and cleaned up the rest.

Discussion: The discussion starts with the main findings that address the author’s hypothesis but then the rest of the discussion is broken up into sections discussing each environmental factor. It would help the reader to have sub-headings that specifically address each hypothesis, especially since many environmental facts had no effect on runner pacing.

**Answer:** We agree with the expert reviewer and have added the relevant subheading to the manuscript.

**Reviewer #2:**

This is an interesting study examining historical finishing times at the Berlin Marathon and associations with weather and environmental factors. The authors should be commended on their handling of such a large data set.

I have a few comments that may be helpful.

Title: Use of ‘age-grade’. Why this term specifically? Is this a common term in the running community? The inclusion for the participants needs further clarification in the methods section. If elites weren’t used what was the cut-off for this? If age-grade is the term, the participants are then not split by age.
**Answer:** Thank you for pointing out our error. We have removed the term from the manuscript. Regarding the cut-off point, we have included all records to get the most generalized results possible.

L110 – Is the greater decline in faster runners a result of trying to compete for the win and therefore possibly not following their pacing strategy?
**Answer:** Indeed, the mismanaging of the set pacing strategy would result in a greater decline. However, it is unknown what percentage of runners have been impacted by their own ambition and how many of them just didn’t have a planned pacing strategy. It could, however, be a topic of a future study.

L140 – methods – subjects – see earlier comment. I think more is needed in this section. Also presumably some runners participated in multiple years. Did you consider analysing this data as a subset to compare the effect of environmental conditions? I appreciate there will be lots of limitations with this but it may be a consideration.

**Answer:** We regrettably did not take into consideration the fact that some runners may have taken part in different editions of the race. Given the large sample size and the fact the race records were registered throughout a 20-year period, we did not think it would be too relevant.

L149 – Is there a way to measure sunshine in intensity i.e W/m2?
**Answer:** Regrettably, we did not have data related to sunshine intensity. The weather stations data of the provided 'Deutscher Wetterdienst' only the sunshine duration (https://opendata.dwd.de/climate_environment/CDC/observations_germany/climate/hourly/sun/DESCRIPTION_obsgermany_climate_hourly_sun_en.pdf).

L230 – Were these variables measured to the degree of accuracy suggested by the number of decimal places?

**Answer:** No. The original, hourly values of the weather variables are provided as integers. The decimal places only appear as a result of the calculations of mean, std and the other statistical values.

Figure 4 – Is it possible to have a minimum, maximum and average value for precipitation? Was it noy just one value?
**Answer:** It is possible, given the weather data is provided hourly (so, for instance, on a given day, we could experience 0 mm of precipitation in the first hour, 1 mm in the second hour, and 2 mm in the third hour, with no further rain during the remaining hours. So we would have min=0, average=3/8, and max=2 in an hour. Nonetheless, we have decided to remove this chart, as the sunshine and precipitation variables are not aligned with the new cumulative values used in the boxplots.

Fig 6 – For sunshine, would you noy expect to see higher exposure with slower runners if measured in minutes? The slower runners would have been exposed to all of the sun for the faster runners and additional sun?
**Answer:** Thank you for pointing this out. We have fixed the issue and we can indeed see from the boxplots that slower runners get longer sun exposition. As a result of the preceding changes this is now Figure 5.

Fig 7 – I appreciate different colours for males and females but using the same colour scheme may help to visually compare the strength of each relationship.

**Answer:** Thank you for the hint. We have changed it to the same color scheme. As a result of the preceding changes, this is now Figure 6.

Discussion – Is there a difference in sweat rates between faster and slower runners? I.e greater sweat rates/ more efficient. Plus increased metabolic heat production?
**Answer:** Sweat rates are highly dependent on each individual physiology and we did not have any data from which we could deduce the sweat rates per runner.

L429-438 – I wonder how relevant this is to the study?
**Answer:** Thank you for pointing out our musings. We have removed the paragraph to compress the study to more relevant information.

L459 – Is this line correct? Does it contrast with L440-441?

**Answer:** That line should be correct by our understanding. Decreasing humidity was associated with a decrease in the pace and running performance.
