# Supplementary material for: Associations between environmental factors and running performance: An observational study of the Berlin Marathon
Source: PLoS One. 2024 Oct 16;19(10):e0312097. doi: 10.1371/journal.pone.0312097 (PMC11482731; doi:10.1371/journal.pone.0312097)
Supplement: Attachment — Submitted filename: PONE-D-24-15986_Revision 2_Comments_31-08-2024.docx [file pone.0312097.s002.docx]

Reviewer #1: Thank you for considering my suggestions but I do not feel they have all been properly addressed.

Thank you for revising the discussion. It is much easier to follow and shows how the current work builds upon the previous literature. Although, the introduction could use better refinement as well to set-up the purpose/better align with the hypotheses of the study.

Still my main concerns follow which have not been fully addressed.
*We thank the expert reviewer for his/her comments and tried to improve again*

1) As environmental temperature increases, relative humidity decreases but the total amount of water vapor in the air may not have changed (absolute humidity). You have shown that with increasing marathon times that relative humidity decreases. This is likely a result of increasing temperatures during the day given the early start times and warming temperature as the day progresses AND the longer the slower runners are exposed to this environmental process. So, this is likely more of a correlational result. It is absolute humidity that determines sweat, evaporation and heat stress risk. Please see PMID: 37255302 and PMID: 29368184, PMID: 38695357. This should be acknowledged and properly addressed.
**Answer:** *We agree with the expert reviewer and added in the limitations ‘As environmental temperature increases, relative humidity decreases but the total amount of water vapor in the air may not have changed (absolute humidity). Relative humidity decreased with increasing marathon race times. This is likely a result of increasing temperatures during the day given the early start times and warming temperature as the day progresses and the longer the slower runners are exposed to this environmental process which is likely more of a correlational result. It is absolute humidity that determines sweat, evaporation and heat stress risk’. We added the suggested references of Prof. Ollie Jay. We also added in the conclusions ‘Absolute humidity and changes in elevations should also be considered.’.*

2) The authors conclude that coaches and athletes can use this information to manage their performance? This has not been addressed? How could this information be used. What the authors are presenting is a retrospective analysis of how environmental factors affected pace.
**Answer:** *We agree with the expert reviewer and delete this statement because other variables have a higher influence on pacing and weather cannot be changed on race day.*

3) The authors did not consider course topography and replied that the course is “Flat”. While it may be flat in comparison to other marathons, it does climb ~20 m with a slight uphill from 20-25km and a bigger incline from 25-27km then a downhill from 27-32km. The authors state in line 208 that running speed “stayed almost constant through 15-20 km. at 20 km was where there is a slight uphill.
**Answer:** *We add in the section ‘The race’ the details ‘In detail, it climbs ~20 m with a slight uphill from 20-25 km and a bigger incline from 25-27 km then a downhill from 27-32 km’ and insert in the limitations ‘The course is not entirely flat and the analyses did not include changes in elevation as a potential influence on pacing of the athletes’ to account for this aspect.*

4) All figures do not directly address the hypotheses. For example: Figure 1 and 2 do not address how an environmental factor affects pace. Figure 3, fine. Figure 4. Comparison of pace between genders but does not include how temperature may differentially affect performance. Given that these individuals were binned by finishing time, what does this tell the reader? Figure 5, the first to incorporate environmental factors that each division of finishers is exposed to. Figure 6. Why not include overall finishing time in matrix, that is one of the conclusions.
**Answer:** *Please see answers to comments below where we think we should have most of these figures in the manuscript. We only deleted figure 4. Figure 5 (former figure 6), the big correlation matrix, has been updated to include the average pace of the full race, as requested by the reviewer.* *The main observation is that the average pace for the full race shows positive correlations with the temperature and the minutes of sunshine for both men and women (so these two factors slow down runners).*

*We added the aspect that the average pace for the full race shows positive correlations with the temperature and the minutes of sunshine for both men and women also in the abstract and in the results section. However, the primary aim of the study was the influence on weather on pacing in the runners.*

5) Discussion: While better organized includes a section (line 366-371) that is not relevant to the findings and not 100% physiologically accurate. Additionally, metabolic rate is the driver for heat production (line 361).
**Answer:** *We agree with the expert reviewer and deleted that section since it has no direct link to performance and weather.*

Although not part of my original critique can the authors confirm that the results indicate that running speed (pace) decreases from 5km to 10, 15, 20, 25, 30, 35, 40km in both men and women and that increasing environmental temperatures increase this rate of slowing but more so in the men than women. This should be very explicitly stated as a result.

**Answer:** *We agree with the expert reviewer and pace decreases with the race distance (most likely as a result of fatigue), with the exception of the pace of the final spurt, where the athletes seem to make a final effort. This is what is shown in the following chart:*

*From the big correlation matrixes, the second part of the reviewer´s comment can also be deducted: for instance, the correlations between the pace at each partial split (5K, 10K, etc.) and the temperature are higher for men than for women (this means the variation of 1° Celsius causes a higher variation in the pace of men than that of women). This pattern is similar for the minutes of sunshine, although to a lesser extent.*

Reviewer #2: Thank you for the detailed responses. My comments have been addressed appropriately apart from one.

L230 – Were these variables measured to the degree of accuracy suggested by the number of
decimal places?
Answer: No. The original, hourly values of the weather variables are provided as integers.
The decimal places only appear as a result of the calculations of mean, std and the other
statistical values.

If the original values were provided as integers then standard practice would be to report them to that. Reporting to decimal places suggests that that level of accuracy and precision was there when it wasn't. Please amend in line with this.

**Answer:** *Unfortunately, we cannot agree with the reviewer here. When we calculate average (mean), standard deviation (std) and percentile values from a list of integers, the resulting values will be non-integer in most cases. There is nothing bad with it, and this makes no statement about the accuracy or resolution of the measurement equipment or its readings. Note also these decimals only appear in the calculated values, but not in the maximum and minimum values (as those are original integer readings – i.e. Min of 7.00 and Max of 27.00).*
